# WNT signaling, the development of the sympathoadrenal–paraganglionic system and neuroblastoma

**DOI:** 10.1007/s00018-017-2685-8

**Published:** 2017-10-22

**Authors:** Jürgen Becker, Jörg Wilting

**Affiliations:** Institute of Anatomy and Cell Biology, University Medical School Göttingen, 37075 Göttingen, Germany

**Keywords:** Neuroblastoma, Sympathoadrenal system, Paraganglion, Neural crest

## Abstract

Neuroblastoma (NB) is a tumor of the sympathoadrenal system arising in children under 15 years of age. In Germany, NB accounts for 7% of childhood cancer cases, but 11% of cancer deaths. It originates from highly migratory progenitor cells that leave the dorsal neural tube and contribute neurons and glial cells to sympathetic ganglia, and chromaffin and supportive cells to the adrenal medulla and paraganglia. Clinically, histologically and molecularly, NBs present as extremely heterogeneous, ranging from very good to very poor prognosis. The etiology of NB still remains unclear and needs to be elucidated, however, aberrant auto- and paracrine embryonic cell communications seem to be likely candidates to initiate or facilitate the emergence, progression and regression of NB. The wingless-type MMTV integration site (WNT) family of proteins represents an evolutionary highly conserved signaling system that orchestrates embryogenesis. At least 19 ligands in the human, numerous receptors and co-receptors are known, which control not only proliferation, but also cell polarity, migration and differentiation. Here we seek to interconnect aspects of WNT signaling with sympathoadrenal and paraganglionic development to define new WNT signaling cues in the etiology and progression of NB.

## Neuroblastoma

Neuroblastoma (NB) is a tumor of the sympathetic nervous system that arises in children under 15 years of age with a frequency of 1:5700. In Germany, NB accounts for 7% of childhood cancer cases, but is responsible for approx. 11% of cancer deaths in children [1]. It originates from highly migratory, sympathoadrenal progenitor cells from the neural crest that migrate long distances and form the adrenal medulla, paraganglia, as well as paravertebral and prevertebral sympathetic ganglia (for review see: [2, 3]). It is commonly accepted to refer to NB as an embryonic tumor [1, 3], which becomes evident when looking at the patient’s age. Approximately 45% of NBs are diagnosed at birth or within the first years of life, with a median age at diagnosis of 1.2 years. Even more strikingly, at the age of 10 years, more than 98% of NBs have been presented clinically [1]. Histologically and clinically, NBs present heterogeneously, spanning from poorly differentiated, highly proliferative, metastasizing and chemotherapy-resistant tumors with unfavorable prognosis to localized, well-differentiated, Schwann cell stroma-rich tumors with good response to mild chemotherapy or differentiating agents such as retinoic acid, and excellent prognosis (reviewed by [4–6]).

Classically, NB are staged into five groups (1–4, and 4S) according to localization, lymph node involvement, and metastasis formation [7, 8]. Currently, classification is about to change into a system considering a variety of risk factors, with the aim to facilitate the comparison of different national studies. Here, the risk groups are divided into L1, L2, M and MS [5, 9]. However, both systems emphasize the special significance of tumors in children up to 18 months of age. These NBs are grouped into the 4S or MS stage, respectively, taking into account that despite metastases to skin, liver, and to a low extend, also to bone marrow, these tumors are in most cases well curable by mild chemotherapy and application of differentiating agents such as retinoids [4, 10]. In general, localized tumors in young patients are highly prone to spontaneous differentiation and regression. As only a small percentage of these tumors progresses to metastatic stages, medical intervention is no longer regarded as the first choice, instead observation and waiting (“wait and see”) may be sufficient [11]. The basis for this type of tumor behavior seems to reside in a natural regression of sympathoadrenal tissue starting at 18 months of age [12, 13].

Even though a multitude of molecules have been found to influence NB behavior and may help to predict the outcome, amplification and/or over-expression of the proto-oncogene *MYCN* is the strongest indicator for highly malignant and therapy-resistant NB. Despite initially successful therapy, these patients frequently suffer from relapse, and die because of metastasis formation and resistance to chemotherapy (reviewed by [14]). However, although MYCN amplification is a potent predictor of disease outcome, it affects only about 25% of the patients, illustrating the urgent need for new diagnostic markers and therapeutic targets in NB. Thereby, interdisciplinary approaches combining developmental biology and pediatric oncology of the sympathoadrenal system have been published in the past 50 years, and may still provide novel hints for new molecular targets for diagnosis and treatment of NB [15–20].

## The sympathoadrenal–paraganglionic system

In the embryo, the sympathoadrenal system consists of the sympathetic nervous system, the adrenal medulla and functionally related paraganglia. Unfortunately, the term paraganglion is often also used for glomera, such as the *Glomus caroticum* and the *Glomera aortica*, which are located along the glossopharyngeal and vagal nerves, and represent sensory organs (pCO_2_, pO_2_, pH). They should not be mixed up with the sympathoadrenal–paraganglionic system, despite their common origin from the neural crest [21]. Paraganglia, which are usually located in the retro-peritoneal region are endocrine organs, which increase the embryos cardiac output under hypoxia (e.g., cigarette smoking of mother) by secretion of catecholamines. They usually regress during early years after birth.

- The sympathetic nervous system has two neuronal levels. The preganglionic, the so-called “first” neurons of the sympathetic nervous system are located in the (mainly thoracic) lateral spinal medulla of segments C8–L2/3. These neurons are not involved in NB development, as their synaptic transmitter acetylcholine is not found in NB, and primary NB is never localized in the central nervous system. The “second” neuron is located in the para-vertebral ganglia of the sympathetic trunk, pre-vertebral ganglia, and pelvic sympathetic ganglia. It is referred to as postganglionic, which means the axons of these neurons leave the ganglion towards target organs.
- In the adrenal medulla, chromaffin, adrenergic (approx. 75%) and noradrenergic (approx. 25%) cells are innervated by pre-ganglionic cholinergic neurons [16]. In addition, the adrenal medulla hosts small groups of post-ganglionic neurons, as well as glia-like cells (sustentacular cells), and may therefore be considered a dual sympathetic ganglion [22–25]. Interestingly, this is the location where the majority of primary NBs develop [3].
- Post-ganglionic sympathetic neurons use noradrenalin as their transmitter, the closely related chromaffin cells produce (nor)adrenalin for endocrine purposes. The only exception are the sympathetic axons that supply the dermal sweat glands, which are cholinergic [26]. Homovanillic acid and vanillylmandelic acid are degradation products of catecholamines, and they are commonly found enriched in the urine of NB patients [27].

**Fig. 1:**
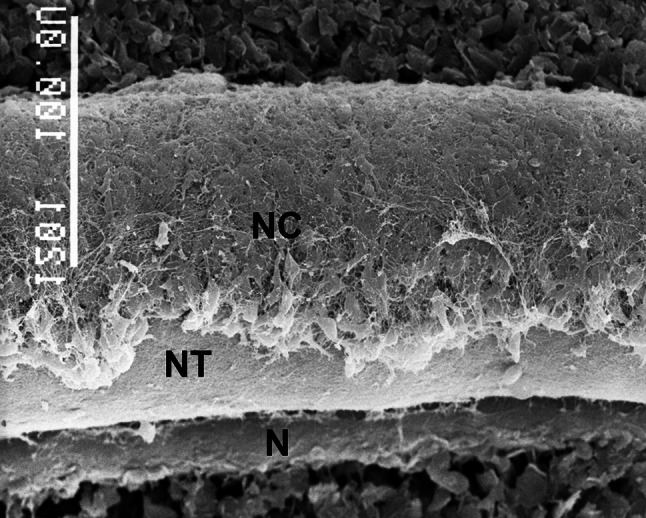
Scanning electron microscopic image of a 2.5-day-old chick embryo, showing neural crest cells (NC) on the dorsolateral surface of the neural tube (NT).* N* notochord (chorda dorsalis). Bar = 100 µm. Republished from [123] with permission; license no.: 4179401141501

These facts, together with further molecular hints that will be discussed later, point out to the sympathoadrenal–paraganglionic origin of NB. During embryonic development, the precursor cells of the sympathoadrenal system delaminate from the dorsal neural tube and form the neural crest (Fig. 1).

## Embryonic development of the sympathoadrenal–paraganglionic system

**Fig. 2:**
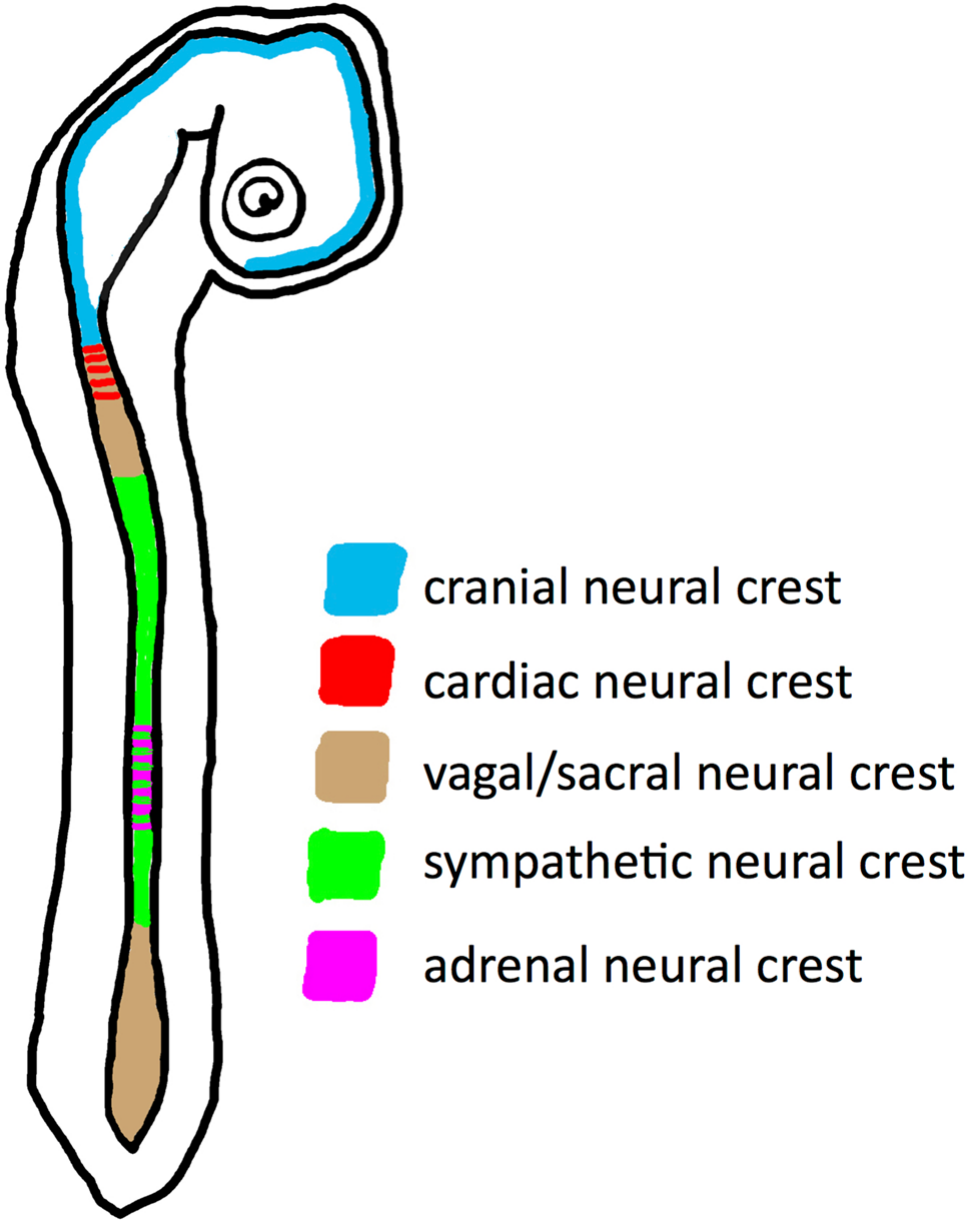
Schematic illustration of the differentiation potential of neural crest cells along the craniocaudal axis of the embryo, as indicated by different colors

Regarding NB as an embryonic tumor, it is of interest that the progenitors of postganglionic sympathetic neurons and chromaffin cells emigrate from the neural crest (NC). Along the craniocaudal axis, the NC can be subdivided into different areas: cranial, cardiac, vagal, sympathetic, adrenal and sacral (Fig. 2) [28, 29]. Sympathoadrenal progenitors develop in specific trunk regions, and are often referred to as trunk NC. The cranial NC as well as cardiac, vagal and sacral parts do not contribute to the sympathoadrenal system. Of note, the trunk NC cells not only form sympathetic neurons and chromaffin cells, but also glial cells, as well as sensory neurons of the dorsal root ganglia, and melanocytes [30, 31]. For the cranial NC a role for WNT signaling has clearly been shown, however, the developmental potential of the cranial NC differs significantly from the other areas by forming connective and skeletal tissues [28, 29, 32].

**Fig. 3:**
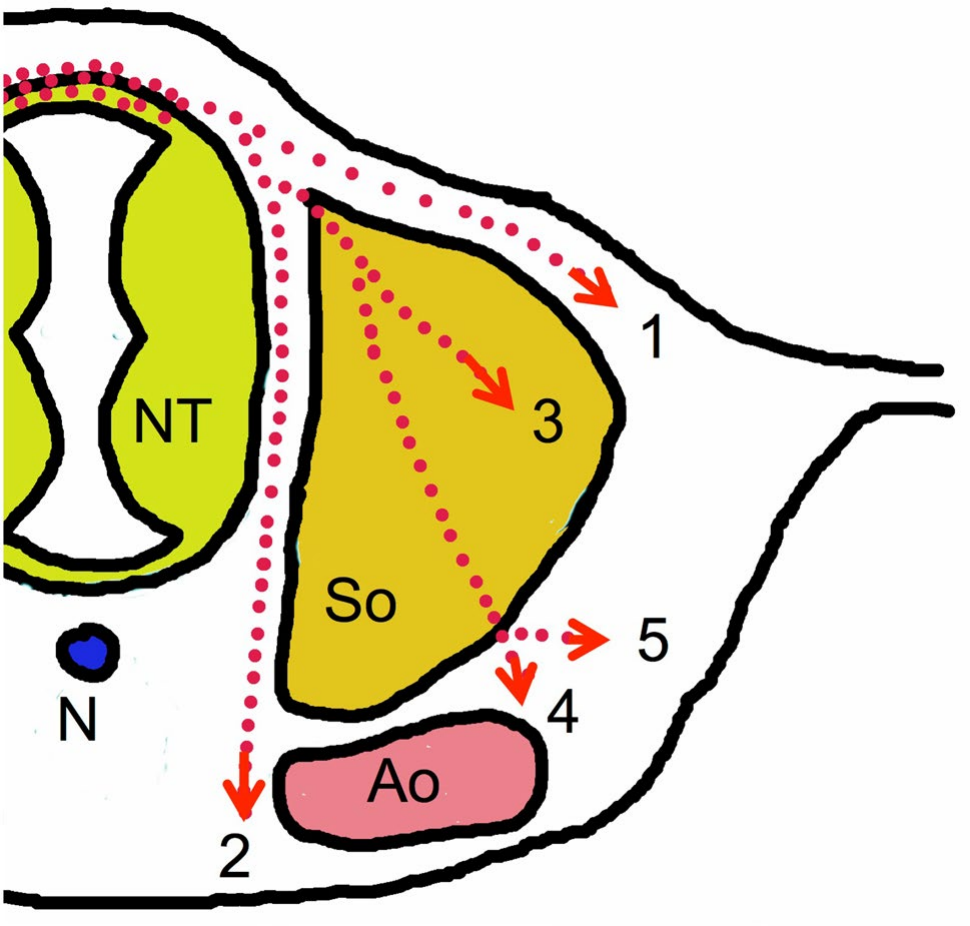
Migration pathways of trunk neural crest cells. (1) Dorsolateral pathway of melanocyte and Merkel cell progenitors. (2–5) Ventral pathways of: progenitors forming prevertebral sympathetic ganglia (2), progenitors of the dorsal root ganglia sensory neurons and glial cells (3), progenitors forming paravertebral sympathetic ganglia (4), and progenitors colonizing the adrenal medulla (5). *Ao* dorsal aorta, *N* notochord, *NT* neural tube, *So* somite (note that at the time of neural crest cell migration, left and right anlagen of the dorsal aorta have not yet fused)

It still is not completely understood how the fate of NC cells is determined. Some of them may already be pre-determined when they leave the neural tube, however, differentiation is also regulated by environmental signals the cells receive during their migratory route (recently reviewed by [33]). There are two major pathways NC cells can take (Fig. 3). The first, called the dorsolateral pathway, enables cells to migrate between epidermis and dermal mesenchyme. Cells following this route will finally invade the epidermis and hair follicles to become melanocytes. This pathway has been shown to depend on WNT signals [34]. The second route is called the ventral pathway. This pathway is further subdivided into two branches; one directed between the somites and the neural tube straight towards the ventral side of the aorta, where the cells differentiate and finally give rise to pre-vertebral sympathetic ganglia. The second branch leads the cells through the anterior (cranial) half of the sclerotome of each somite. Repulsive proteins such as ephrinB1/EphB2 and semaphorin-3F are expressed in the posterior (caudal) sclerotome halves preventing NC cells with appropriate Eph- or neuropilin-2-receptors on their surface from entering [35, 36, 37]. In the anterior sclerotome, thrombospondin is expressed, which allows and promotes NC cells to enter this compartment [38]. Some of the immigrating cells differentiate within the sclerotome and become sensory neurons and glial cells of the dorsal root ganglia. Others make their way through the anterior sclerotome and head on to the dorsolateral part of the aorta, where they give rise to sympathetic neurons and glial cells of the para-vertebral sympathetic trunk ganglia, but also to parasympathetic ganglia and enteric neurons depending on their position along the craniocaudal axis [39].

It has still remained elusive how the different NC cell populations are determined. Most likely, environmental interactions both during migration and in the target organ are of great importance. Exchange of sympathetic and vagal NC has revealed that the emigrating cells are capable to adapt to their new environment, and can produce both cholinergic parasympathetic and adrenergic sympathetic neurons depending on the environment [30, 31, 40]. Thereby, the fate of NC cells is determined by signaling molecules; however, these molecules vary between species [33]. Cells stopping in the sclerotome to develop into glia and neurons of the dorsal root ganglia express WNT and neurotrophin receptors, and may already be pre-determined by WNTs and neurotrophin-3 (NT3) from the dorsal neural tube. Within the sclerotome, the ratio of Delta and Notch expression becomes relevant. Higher Notch expression leads to glia formation, while sensory neuronal precursors are characterized by higher Delta expression. Sympathoadrenal precursor cells that continue their way through the sclerotomes to form sympathetic ganglia and the adrenal medulla do not express receptors for WNTs, NT3, Delta or Notch [41, 42]. Therefore, the transient absence rather than the presence of WNT signaling seems to correlate with their normal development. Their differentiation is dependent on the exposure to bone morphogenetic protein (BMP) from the dorsal aorta, which induces sympathetic neuronal fate. Cells that finally reach the anlage of the adrenal gland enter the adrenal medulla. Migration is induced and guided by BMP from the adrenal cortex, and over-expression of the BMP-binding molecule Noggin in the adrenal cortex inhibits this migration. Upon arrival, their differentiation into sympathetic neurons is blocked by the exposure to glucocorticoids (cortisol) from the adrenal cortex, and they form (nor)adrenalin-producing chromaffin cells [31, 39, 43]. When adrenal cortex is ectopically induced in the embryo by the over-expression of SF1/ZFM 162 (splicing factor 1/zinc finger protein 162), adrenomedullary precursors are attracted to this site [44, 45].

Beside chromaffin cells, in the adrenal medulla there are additional cell populations of sympathoadrenal neural crest origin. Even though their function has not been finally elucidated yet, these are multipolar sympathetic neurons that may regulate blood flow in the adrenal medulla, ‘sustentacular cells’ with glial characteristics, and small cells with a mixed adrenergic/noradrenergic character, which might be chromaffin stem cells [46]. So far there is little evidence for WNT signaling during early embryonic sympathoadrenal development, and rather the absence than the presence of WNTs seems to determine sympathoadrenal progenitor fate.

## WNT signaling

WNT signaling is a crucial and highly conserved pathway in the development of vertebrates as a whole, and specifically also for the development of the peripheral nervous system. In the human, 19 WNT ligands are known so far, which are secreted in a distinct temporal and spatial order, thereby regulating cell proliferation, survival, migration, polarity, and the fate of stem cells [47, 48]. The large numbers of membrane-bound receptors and co-receptors, as well as soluble receptors, which have been identified by now, generate  high complexity of the signaling pathway (reviewed by [49]).

WNT ligands and receptors address several downstream signaling cascades, which are commonly divided into the β-catenin-dependent (also called ‘canonical’) and the two β-catenin-independent pathways, the planar-cell-polarity (PCP) and WNT–Ca^2+^ pathway. WNT1, -2, -3 and -8 are supposed to act in the β-catenin-dependent pathway(s), whereas WNT4, -5 and -11 are regarded as activators of the β-catenin-independent pathways. However, WNT ligands were also shown to act via interconnected intracellular networks, depending on the cellular context [50–52]. In addition, the cellular repertoire of Frizzled (FZD1-10) receptor-family members with divers co-receptors seems to determine, which of the WNT signaling branches are activated. Roughly, interaction of WNTs with FZD members and LRP5 or LRP6 co-receptors addresses the β-catenin-dependent pathway, interaction with ROR-1 and -2 co-receptors only activates PCP, whereas RYK co-receptor may act in all three branches, while PTK7 affects PCP and β-catenin-dependent signaling cascades. Even though the division into three distinct pathways initially suggested independence of each pathway, it is becoming increasingly evident that WNT signaling is not linear and does not fit in this ‘simple’ scheme [53–55]. Instead, some WNT ligands can bind to more than one receptor, and several receptors bind more than one WNT ligand. Additionally, downstream signaling cascades share key switches, thereby influencing each other and building a dense meshwork of signaling routes that allow spatial and temporal specification of cell fate by variable interactions between the three main pathways (reviewed by [47, 56]).

Another obstacle in understanding WNT signaling is the fact that some ligands and receptors can substitute for each other. This may provide a safeguard of the system or serve as a potent tool to ensure a precise temporal and spatial regulation of WNT-driven activations during development, by addressing different promotor structures for the same (or similar) cellular behavior. However, deregulation of WNT signaling is the cause of various types of cancer (reviewed by [48, 57, 58]), but also neurodegenerative diseases such as Alzheimer’s and Parkinson’s disease, and WNT pathways are therefore receiving increasing attention in the overlapping fields of oncology and neurosciences [59–61].

## WNT signaling and the neural crest

In vertebrate embryos, the formation of the NC consists of at least two crucial steps. The first one is the induction of NC cells during neurulation in the neural plate. This initiation requires FGF, retinoic acid and WNT signals (for review see [62]). The second major event is the maintenance of NC cells during neurulation of the embryo by BMP and WNT signaling. Transcription factors such as Snail2, FoxD3, Sox9/10, Twist, cMYC, and AP2 are highly constant markers for NC cells from the time of their epithelio-mesenchymal transition (EMT), throughout their migration phase, and during their differentiation. Inhibition of either Snail or Rho expression disturbs the development of the NC [63–65]. Most likely, Wnt11 non-canonical signaling induces small GTPases such as Rac1 and RhoB. Both promote actin rearrangement, leading to lamellipodia and growth cone formation (Rac1), or induce stress fibres and stabilization of focal adhesion contacts needed for migration [66–70]. Trunk NC cells that form the dorsal root ganglia express neurotrophin and WNT receptors, and respond to NT3 and WNT from the dorsal neural tube [41, 42]. There is evidence that WNT signaling is also involved in NC cell migration into more ventral regions of the embryo. However, development of the sympathetic trunk ganglia seems not to be driven by WNT signals in the first instance. Sympathetic neuron development and formation of ganglia is, as mentioned above, rather induced by BMPs from the dorsal aorta (reviewed by [30, 71]). In mice, BMP induces the expression of transcription factors such as MASH1 and PHOX2A, and thereby converts sympathetic neuroblasts into mature sympathetic neurons. There is, however, evidence that the Fzd3 receptor may be involved in the expansion of neuroblasts and keeps them in a premature proliferative state, but ligands and signaling cascades have not been identified yet, except that β-catenin seems not to be involved [72].

**Fig. 4:**
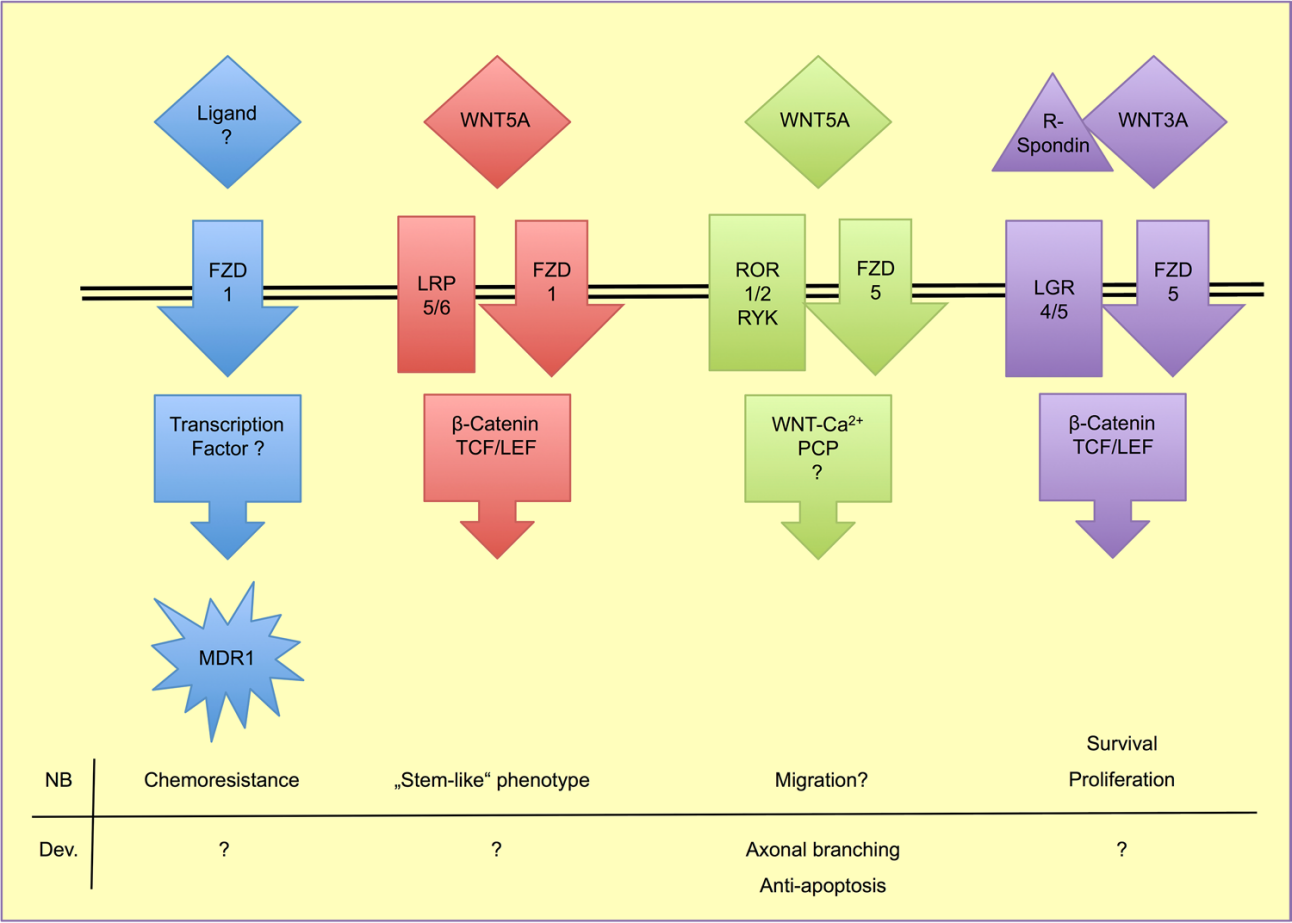
WNT signaling options in NB and sympathoadrenal development

After they have reached the postmitotic stage, sympathetic neurons start expanding their axons toward the innervation target. Molecules involved in this step are hepatocyte growth factor, Artemin, NT3, and nerve growth factor (NGF). Notably, TRK-A, the receptor for NGF, is a favorable prognostic marker in NB ([73, 74]. However, these neurons still lack the key sympatho-adrenergic enzymes tyrosine hydroxylase (TH) and dopamine beta-hydroxylase (DBH), which are induced when the axons reach their target organs. NGF, released from the target tissues, binds to TRK-A on the axonal membrane and induces DBH, TH, and WNT5A transcription in ganglionic neurons [75]. NT3, also known as a TRK-A ligand, cannot substitute for NGF in this case. Endogenous WNT5A then promotes axon branching and subsequent innervation of multiple target cells in an autocrine manner (Fig. 4). Failure of target innervation due to loss of NGF, TRK-A, or WNT5A leads to increased apoptosis and loss of sympathetic neurons [75–77]. Only recently, a similar function of Wnt5a has been shown for the dendritic branching of Purkinje cells in the cerebellum of mice [78]. The induction of target innervation by WNT5A has been shown to depend strongly on the presence of ROR tyrosine-kinase receptors, as ROR1/2 neutralizing antibodies inhibit target innervation in mice [79]. Application of WNT5A to cultured mouse sympathetic chain ganglia neither affected β-catenin-dependent WNT signaling nor JNK- and c-Jun-dependent PCP signaling, but induced strong phosphorylation of PKC, a key player in the Ca^2+^-dependent WNT pathway [75]. These findings imply that WNT5A may signal via ROR1/2 co-receptors and finally affects the WNT–calcium pathway.

However, in vertebrates, Wnt5a/Ror1/2 signaling widely overlaps with the PCP pathway, while Wnt5a/Ryk can be involved in both PCP and WNT–Ca^2+^ signaling (reviewed in [49]). Our expression analyses of 25 NB cell lines show that either ROR1 or ROR2 is found in virtually every cell line, and RYK in fact is expressed in every cell line tested, strengthening the hypothesis of an involvement of ROR1/2 and RYK in sympathoadrenal development and differentiation (Table 1). However, RYK signaling is somewhat obscure, as its tyrosine-kinase domain seems not to be active in signaling, and its extracellular domain is similar to the soluble extracellular WNT inhibitory factor (WIF). Therefore, RYK may (1) act as a decoy-receptor for WNTs, (2) function as co-receptor in concert with other receptors, or (3) may bind WNTs to the cell surface to make them accessible for other receptors. The functions of ROR and RYK were recently reviewed in detail by Green and coworkers [80].

**Table 1:** Expression of WNT pathway molecules in NB cell lines (NBCL)

NBCL	1	2	3	4	5	6	7	8	9	10	11	12	13	14	15	16	17	18	19	20	21	22	23	24	25
mRNA	CHLA-20	CHLA-90	CHP-100^a^	CHP-134^a^	Gimen	IMR-32^a^	IMR5^a^	SMS-KCN	Kelly^a^	LAN 1^a^	LAN 2^a^	LAN 5^a^	LAN6^a^	NB 69	NBL-S^a^	NGP^a^	NLF^a^	NMB^a^	SH-EP	SH-IN	SH-SY5Y	SK-N-AS	SK-N-MC	SK-N-SH	SMS-KAN^a^
WNT5A	**1.0**	0.8	171.2	1.6	6.7	0.4	5.3	2.3	0.8	0.8	3.4	2.1					0.7					3.6		58.4	1.4
WNT5B	**1.0**	0.8	8.2	3.4	14.3	19.0	1.6	1.0	12.0	0.6	2.4	9.7		1.0	0.1	0.8			0.4	2.5	0.9	0.3			1.8
WNT11	**1.0**		2.1	0.4		18.4		0.4	46.2		8.8	117.9	0.4	0.6	1.9	2.4		7.6		0.7			2.5	0.4	1.4
FZD4																									
FZD5	**1.0**	4.7	2.1	0.5	1.4	0.6	1.7	4.3	61.0	0.3	5.2	4.3	155.0	0.6	9.5	1.2	2.7	12.2	1.0	2.7	0.6	1.8	5.3	0.6	0.9
FZD6	**1.0**	1.4	0.8		1.8			0.1	1.9	0.1	1.3	0.5	0.7	0.1	0.8	0.4	0.1	1.3	1.2	2.5	0.5	0.3	0.8	6.6	1.1
FZD3	**1.0**	2.0	0.4	0.4		0.8	2.7	0.2	2.1	0.6	0.1		0.3	0.4	0.8	0.5	0.4	0.3	0.1	0.6	0.8	0.2	0.7	0.5	4.2
ROR1	**1.0**	1.1	2.6	0.2		0.5	0.5	1.2	1.0	0.8	0.4	0.3	0.2	0.7	0.4	0.4	0.6	0.1	0.7	4.1	1.8	0.1	0.8	1.5	0.9
ROR2			0.4			**1.0**				2.5					1.5	1.5				0.8	0.7				2.3
RYK	**1.0**	1.2	1.5	1.1	0.7	1.3	4.3	0.6	3.6	0.9	2.6	1.1	1.7	0.6	0.9	1.6	0.8	1.6	0.7	1.6	1.2	1.4	1.1	1.5	3.0
LRP5	**1.0**	0.5	1.9	1.2	0.1	1.2	1.4	0.5	1.0	0.8	0.5	1.3	0.4	0.4	0.5	1.5	0.1	1.1	0.6	0.5	0.6	0.3	0.6	0.3	1.0
LRP6	**1.0**	0.8	1.8	0.3	0.3	1.0	1.7	0.4	3.2	0.4	0.2	0.3	0.8	0.4	0.6	0.5	0.5	0.9	0.2	1.0	0.5	0.3	0.4	0.4	0.8
VANGL2	**1.0**	1.3	0.1	0.4		0.9	1.4	0.2	0.5	0.3	0.3		0.1	0.2	0.2	0.5	0.1	0.1			0.1	0.1		0.1	0.2
PTK7	**1.0**	1.5	1.0	1.0	0.4	2.6	1.0	1.5	1.7	1.6	1.0	2.1	1.0	1.2	1.2	3.5	0.7	2.9	0.5	1.9	2.5	2.0	6.1	1.8	0.4
IGF2BP1	**1.0**		2.4	3.5	0.3	5.0	16.5	0.3	2.6	0.2	0.3	0.1		0.7	0.7	0.5	0.3	0.3	0.3				0.6		0.1
LGR5	**1.0**		2.3							1.7					2.0					5.4		1.6			
LGR4	**1.0**	0.3	0.4	1.4	0.3	1.0	0.9	0.3	0.7	0.1	1.3	0.2	0.1	0.2	0.6	0.6	0.2	0.2	0.2	0.4	0.3	0.3	2.9	0.1	1.1
DKK1	**1.0**	1.4	0.4	2.9	1.3	1.7	0.1	0.1	0.3	12.7	0.7	0.8	16.0	7.4	94.1	110.8		0.2	4.1	6.6	6.9	5.2	0.0	1.3	26.3
DKK2	**1.0**		0.1		0.6	0.1				0.5	3.5									91.1	1.3	0.1		89.0	0.1

Expression of indicated transcripts was measured by real-time RT-PCR and calculated as relative expression values according to the ΔΔC_T_ method as described previously [124]. CHLA-20 was chosen as reference cell line (= 1, bold) for all transcripts, except for ROR2 where IMR32 was chosen. Empty fields represent not detectable transcript levels

*The following primers were used (5′-fwd/rev-3′)*: *DKK1* gcaccttggatgggtattcca/gcacagtctgatgaccggag, *DKK2* cccagtacccgctgcaataa/cgatctctgtgccgagtacc, *FZD3* tgaccaacagacagcagctt/acaaagaaaaggccggaaat, *FZD4* gacaactttcacaccgctca/tgcacattggcacataaaca, *FZD5* ctggggactgtctgctcttc/gacggttagggctcggatt, *FZD6* tgggtctctgatcattgtcg/ttctggtcgagcttttgctt, *IGF2BP1* tagtaccaagagaccagacc/gatttctgcccgttgttgtc, *LGR5* ggaaacctctccagcttggta/tctaggctgtggagcccatc, *LGR4* acccagtgaagccattcgag/gtcctcggggactgaggtaa, *LRP5* gacaacggcaggacgtgtaa/agatcctccgtaggtccgtc, *LRP6* ctccggcgaattgaaagcag/taagtcccacaggctgcaag, *PTK7* ctgcagtggctctttgagga/gttggcaaacactgtggctc, *ROR1* caacaaacggcaaggaggtg/atcctggacttgcagtggga, *ROR2* gaccctttaggaccccttga/ggccttggacaatggtgata, *RYK* agttcgttggatggctcttg/gagttcccacagcgtcactc, *VANGL2* ctgtctacaaccctgccctc/ggtgctgttttcctctccga, *WNT5A* atgaacctgcacaacaacga/ccagcatgtcttcaggctac, *WNT5B* ttctgacagacgccaactcc/ggctgggcaccgatgataaa, *WNT11* ctgcagagctcacctgactt/gttgcactgcctgtcttgtg

^a^MYCN-amplified cell lines

## WNT signaling in neuroblastoma

So far, WNT signaling in NB has been a neglected topic, and only few studies have provided evidence for the impact of WNTs in NB, and demonstrated that the dissection of WNT signaling into the so-called canonical and non-canonical pathways may not properly reflect the heterogeneity of the lesions (Fig. 4). WNT pathway studies in NB were first conducted in the mouse cell line Neuro2a in the context of neurite outgrowth and differentiation [81, 82]. Although the studies added new and valuable information on WNT pathway mechanisms, they regarded NB cells as a neuronal model system, and not as a malignancy model. However, they provided evidence for WNT signaling in NB. The first evidence for WNT signaling in human primary NB was published in 2005, when Blanc and co-workers observed that local xenografts of human IGR-N-91 NB cells in mice exhibited higher WNT5A expression than bone marrow and cardiac metastases of the grafted cells [83]. They also found increased WNT5A mRNA levels in primary human NB samples with favorable outcome. As mentioned above, Wnt5a is important for the terminal differentiation of sympathetic neuroblasts after target innervation, and failure in this signaling pathway might be causal for NB development in early childhood. Notably, Dyberg et al. reported that high expression of *VANGL2* and *PRICKLE1* correlates with low-risk NB and reduced levels of active β-catenin [84]. Both *VANGL2* and *PRICKLE1* are key players of the (non-canonical) PCP-pathway. Inhibition of ROCK1/2, central molecules of PCP, increases PRICKLE1 expression and inhibits β-catenin pathway activity. This suggests a direct inhibition of β-catenin-dependent WNT signaling by PCP via *PRICKLE1*. Another recent publication focused on transcriptome analyses and computational interaction studies in NB, melanoma and colorectal carcinoma, and provided data that correlate high WNT3A, WNT5A, APC, or FZD10 expression with longer event-free survival, while high WNT3 or FZD1 indicates less favorable outcome [85]. This group also pointed out to interactions between WNT signaling and *MYCN* activity at various levels, and provided a gene signature based on the WNT pathway, which may improve risk stratification for NB patients.

Liu and co-workers were not able to confirm differential expression of WNT5A in high- vs. low-risk NB, but found higher β-catenin levels and more nuclear β-catenin in *MYCN* non-amplified cell lines [86]. In our expression analyses, WNT5A was detectable in 16 out of 25 cell lines and there was no difference between *MYCN*-amplified and *MYCN*-non-amplified cell lines (Table 1). However, the cell lines are commonly derived from progressed NB or isolated from metastatic sites [87]. Interestingly, every single cell line we tested expressed at least one of the WNT species that can act in the β-catenin-independent pathways: WNT5A, WNT5B, or WNT11. On the other hand, our data reveal a tendency for higher LRP5 or LRP6 expression in *MYCN*-amplified cell lines, which may enhance β-catenin-dependent signaling (Table 1).

FZD5 and -6 are considered as receptors for WNT5A that act preferentially in the β-catenin-independent pathways. In several mouse and human NB cell lines, a subpopulation of FZD6 expressing cells was shown to exhibit a highly tumorigenic ‘stem-like’ phenotype that disappears in FZD6-siRNA knock-down experiments [88]. Thereby, FZD6 expression correlates with activation of non-canonical WNT signaling, as shown by phosphorylation of JNK (jun kinase).

These apparently conflicting results may be due to the genomic heterogeneity of NB lesions and cell lines. Another explanation is provided by Duffy and coworkers. They observed that NB cells depend on a strictly balanced WNT signaling to direct their behavior towards proliferation, anti-apoptosis and metastasis, or differentiation [85]. Imbalance induced by both massive inhibition and massive activation of WNT signaling will induce abnormal cell behavior. This angle of view seems to explain many puzzling findings on NB-WNT signaling provided so far.

## WNT signaling and neuroblastoma therapy resistance

In relapsed NB patients, resistance to chemotherapy is a major problem. There is evidence that FZD1, a WNT receptor dedicated to the β-catenin pathway, mediates the expression of the multidrug-resistance-protein 1 (MDR1) efflux-transporter that confers drug resistance to tumor cells [89]. Knockdown of FZD1 decreases MDR1 expression and restores chemo-sensitivity in resistant cell lines. Also in these experiments, TCF/LEF-mediated activation of target transcription could not be detected, and suggests the existence of an alternative pathway to the transcriptional activation through nuclear β-catenin, probably via ‘developmental’ transcription factors such as SOX and FOXO [89].

Retinoids (RA) are standard drugs used for differentiation therapy in NB [90–92]. However, in *MYCN*-amplified high-risk tumors, differentiation therapy often fails. Very recent expression analyses show that DKK1 and FZD7 are inversely regulated by MYCN and RA, and may be part of the RA-induced differentiation signaling cascade [93]. Failure of differentiation therapy with retinoids may also be due to increased levels of HIF1/2α, which activate β-catenin-dependent WNT signaling, and thereby promote proliferation and metastasis formation [94]. High HIF expression in neuroblastoma cells promotes an undifferentiated state with stem-like phenotype [88, 95], and correlates with poor prognosis [96]. Inhibition of HIF was shown to shift cells into a more differentiated expression profile, and enhance differentiation of NB cells by retinoids [97, 98]. This effect seems to be associated with inhibition of the WNT/β-catenin signaling pathway, or a significant disturbance of the WNT balance in malignant cells.

## Unorthodox WNT signaling in neuroblastoma

The functional homologues LGR4 and LGR5 are G-protein-coupled receptors that bind R-spondins, and thereby amplify β-catenin-dependent WNT signaling [99] (Fig. 4). In HEK293 (human embryonic kidney cell-line), LGR5 is co-internalized with FZD5 upon stimulation with WNT3A [100]. LGR5 is regarded as a marker for tumor stem cells and has been shown to be up-regulated in highly tumorigenic primary NB cells, which are able to form tumor spheres in nude mice [101]. In NB cell lines, co-stimulation with WNT3A and R-spondins strongly activated TCF/LEF reporter-gene constructs, and LGR5 knock-down by siRNA inhibited this reaction [102]. Additionally, the same authors observed induction of apoptosis upon LGR5 knock-down in NB cell lines. Meta-analyses of micro-array data from primary NB revealed increased expression of LGR5 in *MYCN*-amplified tumors, and positive correlation with adverse outcome [103]. Our expression analyses confirm LGR5 expression only in some NB cell lines, whereas in the majority of cells it is completely absent. However, its functional homologue LGR4 could be detected in all cell lines to variable extend (Table 1). Taken together, LGR5 and canonical signaling seem to be of great importance for the expansion of NB from tumor stem cells, and may serve as a therapeutic target in NB.

LGR4/-5 expression is also of interest when considering developmental aspects. As mentioned above, migrating sympathetic neuroblasts require BMP from the dorsal aorta to differentiate into postganglionic neurons during formation of sympathetic ganglia [39, 43]. Moreover, a BMP gradient seems to attract sympathetic precursors to the adrenal medulla [30, 44, 45]. Norrin, an antagonist of BMP-signaling, has been shown to act via LGR4 and FZD4 [104]. Binding of Norrin to LGR5 has also been shown; however, no activation of β-catenin-dependent signaling could be detected. Even though we could not detect FZD4 expression in any of our 25 NB cell-lines, it cannot be ruled out that a similar mechanism involving aberrant LGR5 and WNT signaling may prevent BMP-driven migration and differentiation of precursor cells, leading to ectopic location of poorly differentiated neuroblasts. Such cells seem to be highly sensitive to progress into neuroblastoma, and this may be facilitated by the WNT/β-catenin-enhancing properties of LGR4/-5–R-spondin signaling. Other groups, using RNA-seq, found FZD4 readily expressed in NB cell-lines [85, 105]. This discrepancy is puzzling, and further careful studies have to reveal the status of FZD4 expression at RNA and protein levels.

## Chromosomal aberrations in neuroblastoma affect WNT signaling

NBs frequently present with chromosomal aberrations. Thereby, loss of genetic material can be observed at chromosomes 1p and 11q, whereas gain of genes can be found at chromosomes 11p and 17q [4, 14]. A recent study revealed that loss in 1p and 11q is more frequently found in metastases and relapsed tumors than in primary tumors, underlining the importance of the two chromosomal locations for NB [106]. They and others found mutations accumulating in *ALK*, and especially in the RAS/MAPK-signaling pathway [107]. However, this does not exclude that the complete loss of WNT pathway genes in 1p or 11q may also be of relevance for NB etiology. We used a gene database search to identify prominent members of the WNT signaling pathway [108]. Interestingly, there are a number of candidates in the affected areas that support the involvement of developmental control genes in NB (Table 2). WNTs such as WNT4, WNT2B and WNT11 are located in regions where loss of chromatin is observed, and WNT receptors such as FZD4 and ROR1 are also located there. In particular, FZD4 being located in a region where chromosomal loss is often observed could provide an important clue to NB genesis. None of the cell lines we screened showed detectable mRNA levels (Table 1). As mentioned above, this seems to be in conflict with recently published RNAseq data, and further studies should pay special attention to this discrepancy [85, 105]. However, we speculate that loss of functional FZD4, either by mutation or chromosomal loss could be a common feature of NB cells and a key event in NB genesis.

**Table 2:** Correlation between developmental genes with chromosomal aberrations in NB

Common aberration in NB:	Deletion		Gain	
Locus	Chr. 1p	Chr. 11q	Chr. 11p	Chr. 17q
WNT pathway	WNT2B	WNT11	DKK3	FZD2
	VANGL1	FZD4	LGR4	WNT3
	DVL1			WNT9B
	WNT4			
	ROR1			
	WLS			
Sympathoadrenal development	EphA2, -A8, -B2, -A10	PHOX2A		NGFR
	NGF	ROBO3, -4		NOG
	Notch2			

Genes of interest were picked from the “Atlas of Genetics and Cytogenetics in Oncology and Haematology” [108]

Furthermore (disregarding WNT2B and WNT9B: for both of which the specific Wnt pathway, which they activate, has not yet been clearly resolved), there may be a shift in WNT signaling from β-catenin-independent signaling ligands (WNT4A and WNT11), which are located in loss-prone regions to β-catenin-dependent signaling (represented by WNT3) on the gain region. This may be enhanced by increased expression of LGR4 as discussed above [99] (Fig. 4). Decreasing expression of β-catenin-independent signaling molecules ROR1 and VANGL1, which are located in loss-prone regions, may also contribute to this shift. However, DKK3, which is also located in a gain location and is an inhibitor of β-catenin-dependent signaling, does not fit perfectly into this hypothesis. DKK1 and DKK3 were shown to correlate with NB maturation by acting as inhibitors of NB cell differentiation [109, 110]. Both are down-regulated by *MYCN*, most likely via miRNA-mediated mRNA degradation [109–112]. Expression profiling reveals that the homeobox-domain protein MSX1, which is involved in NC development, induces DKK1-3 and SFRP1 in NB, and its expression correlates with good prognosis [113]. However, WNT5A and WNT3 signaling via DVL3 remained active, indicating the puzzling roles for soluble WNT inhibitors in NB.

In addition to WNT signaling, there are other genes on the affected chromosomal regions linked to the development of the sympathoadrenal system (Table 2). Failure of cellular guidance that misleads cells to ectopic locations is one of the key events in the emergence of malignancies. Here, potential up-regulation of Noggin (NOG), which inhibits BMP signaling, may prevent NC cells from participating properly in ganglion formation. Additionally, aberrant expression of Eph-receptors could mislead NC cells on their way through the somites to ectopic sites.

## WNT pathways as a therapeutic target

As discussed above, WNT signaling seems to offer multiple targets for new therapeutic strategies. Thereby, both β-catenin-dependent and independent pathways may be suitable. Very recently, FZD2 has been shown to interact with WNT5A and WNT3A, thereby activating ‘canonical’ and ‘non-canonical’ WNT signaling in NB cells [114]. Knockdown of FZD2 was shown to suppress the growth of mouse xenograft NB and reduce tumor angiogenesis.

WNT/β-catenin signaling is also the focus of drug optimization approaches. Tankyrase1 (TNKS1) is known to phosphorylate Axin1/2, thereby regulating the β-catenin destruction complex. Inhibition of TNKS1 with XAV939 stabilizes this complex and increases β-catenin destruction, which prevents WNT/β-catenin-signaling [115, 116]. The loss of β-catenin reduces NB growth and increases apoptosis (Fig. 4). Another group very recently found increased sensitivity to doxorubicin in SH-SY5Y cells after treatment with XAV939 [117]. Doxorubicin is a standard drug in NB treatment, and resistance a major problem. DKK1 knockdown in cell lines led to increased sensitivity to doxorubicin [118]. In patients with metastatic NB, high DKK1 serum levels correlate with poor outcome, however, there was no difference between all NB patients and healthy controls, or between patients with or without metastases.

Cytotoxic therapy might also be improved by combining chemotherapy with LiCl, an activator of the WNT/β-catenin pathway, as shown in cell culture experiments [119]. Recently, a group demonstrated that WNT inhibitory factor 1 (WIF1) is silenced by hyper-methylation in NB cell lines [120]. Restoration of WIF1 expression inhibited proliferation and caused down-regulation of β-catenin and its transcriptional targets. So far, WNT signaling can be targeted by several drugs, such as porcupine inhibitors that prevent WNT secretion, FZD-receptor blockers or decoy receptors, and modulators of β-catenin stability or its downstream signaling. An overview considering targets, signaling molecules and risk factors of the WNT pathways is provided by recent reviews [57, 59, 121]. Clinical trials of WNT-targeting therapies are underway for different solid tumor entities, and successful passing of these trials will probably make them interesting for NB therapy. Interactions between the MYCN and WNT pathways provide additional interesting therapeutic targets, especially for high-risk patients, which are in great need for new drugs. High-throughput methods will help identify new targets [93, 106, 107, 120, 122].

## Conclusions

According to our current knowledge, WNT signaling only seems to be indirectly involved during early development of the sympathoadrenal system. However, at the stage of direct target innervation, WNT5A and ROR signaling is indispensable for terminal differentiation of adrenergic neurons. Regarding NB, several groups have published (in part conflicting) results, which once more depict the immense heterogeneity of this sympathoadrenal tumor. Nevertheless, the data presented so far strongly suggest a critical role for WNT signaling in NB. Our expression data (Table 1) support the possibility of β-catenin-independent WNT signaling in NB cell lines, as shown earlier [83, 84, 113, 114]. All cell lines we examined express WNT5A, 5B, or WNT11, but expression levels vary dramatically. Also, transcripts for receptors such as FZD5, ROR1, RYK, VANGL2, and PTK7 are present in almost all cell-lines, but in varying amounts. Therefore, all components needed to activate the WNT–PCP and WNT–Ca^2+^ pathways are available. Additionally, β-catenin-dependent WNT pathways were shown to play a pivotal role in NB expansion and progression. Analyses of chromosomal aberrations reveal a possible shift from β-catenin-independent to classical WNT–β-catenin pathways with a possible key role for the loss of FZD4 expression. Both β-catenin-dependent and independent pathways facilitate a broad spectrum of cell–cell interactions, and it is highly likely that there are mechanisms that link the genesis of NB to early developmental processes. The heterogeneity of NB and the complexity WNT signaling are clinically and scientifically challenging. Considering the similarities between the embryonic development of the sympathoadrenal–paraganglionic system and the biology of NB, progression in WNT signaling research in one field may be transferred to the other, which may finally result in new and improved therapeutic strategies for the benefit of the young patients.

## Abbreviations

ALK: Anaplastic lymphoma kinase
BMP: Bone morphogenetic protein
DBH: Dopamine beta-hydroxylase
DKK: Dickkopf (WNT signaling pathway inhibitor)
FOXO: Forkhead box protein *o* transcription factor
FZD: Frizzled (G protein-coupled) receptor
GSK3β: Glycogen synthase kinase 3β
ILK: Integrin-linked kinase
LGR4/5: Leucine-rich repeat-containing G protein-coupled receptor 4/5
MDR1: Multidrug resistance protein 1
NB(s): Neuroblastoma(s)
NC: Neural crest
Neuro2a: Mouse-derived neuroblastoma cell line
NGF: Nerve growth factor
NOG: Noggin
NT3: Neurotrophin-3
PTK7: Protein tyrosine kinase 7
ROR1/2: Receptor tyrosine kinase-like orphan receptor-1/-2
R-spondin-1: Secreted protein, interacts with WNT4
RYK: Related to receptor tyrosine kinase
SFRP1: Secreted frizzled-related protein 1
SOX: Sry-related HMG box transcription factor
TCF/LEF: T-cell factor/lymphoid enhancer factor (transcription factor)
TGFBI: Transforming growth factor-induced protein h3 = Keratoepithelin, Bigh3
TH: Tyrosine hydroxylase
TNKS1: Tankyrase1
TRK-A: Tyrosine (tropomyosin) receptor kinase-A
VANGL2: Van-gogh-like-2
WNT: Wingless-type MMTV integration site family
